# Cyanide Toxicity!! Colour of Blood Says It All

**DOI:** 10.5005/jp-journals-10071-23141

**Published:** 2019-03

**Authors:** Niranjana Panigrahi, Sai Praveen Haranath, Aleem MA, Y Srinivas, Sudeep Sirga, Ramkumar, Sarala K

**Affiliations:** 1,3,5-7 Department of Critical Care Medicine, Apollo Hospital Hyderabad, Telangana, India; 2,4 Department of Pulmonary and Critical Care Medicine, Apollo Hospital Hyderabad, Telangana, India

**Keywords:** Cherry red venous blood, Cyanide antidot, Cyanide poisoning, Profound shock

## Abstract

Cyanide toxicity is a fatal condition if not detected and treated in stipulated time. Lack of rapid detection modalities, and nonspecific nature of clinical presentation make the diagnosis more challenging. Cherry red colour of blood might be the only clue sometimes. We present a case of sudden onset altered sensorium which was detected as cyanide poisoning and treated successfully with antidots on the basis of central venous blood colour and corroborative presentation.

**How to cite this article:**

Panigrahi N, Haranath P *et al*. Cyanide Toxicity!! Colour of Blood Says It All. Indian J Crit Care Med 2019;23(3):155-156.

## INTRODUCTION

Cyanide is a deadly poison, utilized in some pharmaceutical and chemical industries, and is rarely available to the public. It gets rapidly absorbed into the circulation and tissues, and arrests aerobic metabolism which leads to sudden deterioration of organ function and death. Rapid diagnosis and treatment with the antidote is the only way to rescue these patients. Diagnosis is challenging in case of unwitnessed self poisoning, due to lack of rapid detection methods and urgency of the situation. Clinical presentation is often vague and mimics many other conditions with hypoxia or hemodynamic shock. Cherry red blood venous colour which is described in the literature may give clues in some cases who present early. We summarize our case as an unwitnessed sudden onset altered sensorium which was detected as cyanide poisoning on the basis of central venous blood colour and blood gas parameters.

## Case Report

A 36 year female without comorbidities was brought to the emergency room unconscious and in respiratory distress at 1:40 pm. She was found unresponsive around 1:15 pm after having gone to the restroom at 1:10 pm and had been conscious and alert prior to this episode. On arrival at the emergency room, she had a barely palpable pulse and unrecordable blood pressure. She was intubated, connected to the ventilator and resuscitated with crystalloids and noradrenalin through a wide bore peripheral cannula. Arterial blood gas reports at 1:50 pm revealed a severe acidosis of combined respiratory and metabolic origin with lactate 15 mmol/l and bicarbonate 6 mmol/l. Investigations to evaluate sudden onset unconsciousness and shock were initiated with a differential diagnosis including anaphylactic shock, vasovagal episode, massive stroke or substance abuse. Urine toxicology, electrolyte, renal and liver function markers and urgent CT brain was ordered. Central venous cannulation with ultrasound guidance was tried via left internal jugular vein in view of ongoing resuscitation and use of vasopressors. We got bright red blood, which was mimicking an arterial sample, but venous placement of cannula was reconfirmed with a USG and free flow of fluid method and subsequently with blood gas analysis. We suspected a carbon monoxide poisoning or cyanide poisoning. Carbon monoxide poisoning was ruled out with the sudden onset. The family had no knowledge regarding access to substances that can cause cyanide poisoning. Simultaneous venous blood gas analysis from the central vein and arterial blood gas testing revealed similar and high values of SCvO_2_ and SPaO_2_ (92% and 97% respectively). A strong suspicion of cyanide poisoning was made but could not be confirmed because of unavailability of rapid laboratory testing. Around this time the patient started regaining consciousness partially, despite continued severe metabolic acidosis. Upon questioning she confirmed nonverbally, an intentional consumption of substance, and that to be cyanide through. Family was counseled regarding the most probable diagnosis as cyanide poisoning, urgency of situation, time and unavailability for confirmatory tests and the antidotes available. Antidote for cyanide was administered with sequential injection of 3% sodium nitrite 600 mg IV over 3 minutes, followed by 25% sodium thiosulfate 25 g IV over 30 minutes at 2:40 pm. Over next few hours patient started improving in terms of blood pressure, ABG and sensorium with continuation of other conservative treatments. She improved gradually over night and was extubated the next day.

## Blood Gas Analysis

**Fig. 1:**
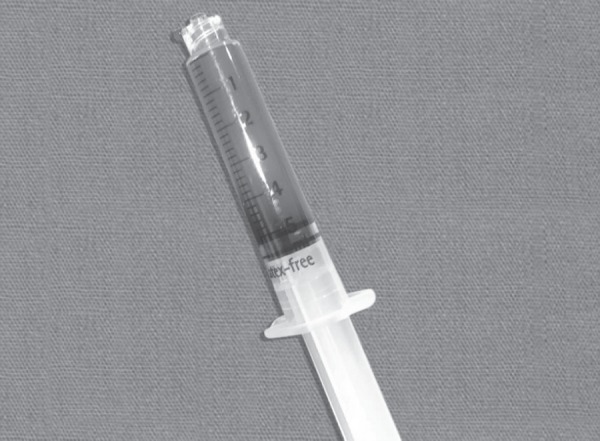
Colour of arterial blood sample

**Fig. 2:**
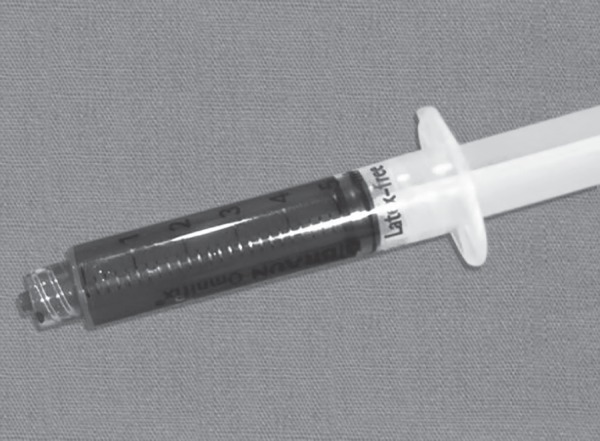
Colour of venous blood sample

Postextubation psychiatric counselling revealed patient was suffering from depression. She obtained cyanide from a water purification plant where she works, it is used for assessment of zinc level in water(x). She was discharged with proper medication and counselling (Table 1, Figs 1 and 2).

## DISCUSSION

Cyanide is a deadly poison, utilized in some pharmaceutical and chemical industries, and is rarely available for public access^1,2^. It's molecular structure consists of a cyano group (a carbon triple-bonded to nitrogen); C≡N in combination with potassium or hydrogen^3^. Once in the bloodstream, cyanide rapidly reacts with ferric ions of cytochrome oxidase *a*, a mitochondrial enzyme responsible for oxidative phosphorylation in the final pathway of cellular respiration. Inhibition of oxygen utilization causes sudden surge of anerobic metabolism^4^. Clinical presentation of acute cyanide exposure varies depending on amount; starting from dizziness to loss of consciousness, cardiac and respiratory failure, hypoxic brain injury, and dose-dependent death within minutes to hours^5^. Unfortunately, there is no test for rapid confirmation of cyanide toxicity, so treatment must be based on a presumptive diagnosis^3^. Cyanide poisoningis treatable when quickly recognized and treated with antidote.

Out of two antidotes, hydroxocobalamin is claimed to have fewer adverse events than the cyanide antidote kit^6^. Understanding both available antidotes and their respective benefits, contraindications is highly essential for intensivists.

**Table 1:** Blood gas analysis

*Time*	*pH*	*PaCO2 (mmHg)*	*PaO2 (mmHg)*	*HCO3 (mmol/L)*	*Lactate (mmol/L)*
1.51 pm	7.170	17	175	6	15
4.30 pm	7.317	39	85	19.8	4.7
7.25 pm	7.325	44.8	98	21	3.1
6.58 am (next day)	7.376	43.3	175	20.8	0.8

### Cyanide Antidote Kit

The cyanide antidote kit has been used for decades for acute cyanide poisoning on the basis of clinical reports of Chen and Rose^6^. This kit consists of 3 medications given together: Amyl nitrite and sodium nitrite is administered intravenously, before sodium thiosulfate. The nitrites convert hemoglobin to methemoglobin which binds to cyanide to form cyanmethemoglobin. Because cyanide appears to bind preferentially to methemoglobin rather than cytochrome oxidase in the mitochondria and frees the enzymes for aerobicmetabolism^7^. Sodium thiosulfate clears cyanide from cyanmethemoglobin to form thiocyanate which gets excreted through kidney.

## CONCLUSION

Hence, in our case, suspecting on the basis of venous blood colour and correlating the clinical scenario was the key to our timely diagnosis. We felt it is not worth waiting for confirmatory test to prove cyanide toxicity. The risk of using the components of the cyanide kit is less than the potential benefits and prevention of death. Our case may be an eye opener to keep a wide differential diagnosis for successful treatment in unwitnessed sudden onset unconsciousness.
